# Short-term effect of ligature-induced periodontitis on cardiovascular variability and inflammatory response in spontaneously hypertensive rats

**DOI:** 10.1186/s12903-021-01885-6

**Published:** 2021-10-11

**Authors:** Aline Barbosa Ribeiro, Thais Marques da Silva, Nilton Nascimento Santos-Júnior, Jaci Airton Castania, Rubens Fazan, Helio Cesar Salgado

**Affiliations:** Department of Physiology, Ribeirão Preto Medical School, University of São Paulo, Avenida dos Bandeirantes, Ribeirão Preto, São Paulo 14049-900 Brazil

**Keywords:** Nitric oxide, Periodontitis, Myocardial inflammation, Heart rate variability, Arterial pressure variability

## Abstract

**Background:**

We previously reported that periodontal disease (PD) induces high arterial pressure variability (APV) consistent with sympathetic overactivity and elicits myocardial inflammation in Balb/c mice. However, it is unknown whether PD can change APV and heart rate variability (HRV) in spontaneously hypertensive (SHR) and normotensive Wistar-Kyoto (WKY) rats. This study aimed to evaluate the hemodynamic level, HRV, and APV associating with myocardial inflammation and plasma concentrations of oxide nitric (NO) in SHR and WKY rats with PD.

**Methods:**

Three weeks after bilateral ligation of the first mandibular molar, or Sham operation, the rats received catheters into the femoral artery and had their arterial pressure (AP) recorded the following day. Subsequently, plasma, heart, and jaw were collected. The NO was quantified by the chemiluminescence method in plasma, and the myocardial IL-1β concentrations were evaluated by ELISA. In the jaw was evaluated linear alveolar bone loss induced by PD.

**Results:**

The linear alveolar bone loss in jaws of SHR with PD was higher than in all other groups. AP and heart rate were higher in SHR than in their WKY counterparts. SHR with PD showed lower AP than control SHR. HRV and APV were different between SHR and WKY rats; however, no differences in these parameters were found between the animals with PD and their control counterparts. Plasma NO and myocardial IL-1β concentrations were higher in SHR with PD as compared to control WKY. A significant correlation was found between linear alveolar bone loss and plasma NO and myocardial IL-1β concentrations.

**Conclusion:**

Our results demonstrated that short-term PD lowered the AP in SHR, which might be due to the higher levels of plasma NO. Even though PD did not affect either HRV or APV, it did induce myocardial inflammation, which can determine cardiovascular dysfunction in long-term PD.

## Background

Hypertension is a severe public health problem due to its high prevalence [1]. It is a multifactorial clinical condition characterized by high and sustained levels of blood pressure, a significant predictor of cardiovascular risk [2]. The mechanisms involved in the development of hypertension include a decrease in heart rate variability, increased blood pressure variability, autonomic imbalance (sympathetic overactivity and deficit in cardiac parasympathetic modulation), endothelial dysfunction (reduction of nitric oxide release), oxidative stress and inflammation [3–5]. Even though relatively recent studies have shown the role of the immune system (innate and adaptive immunity) in the onset of arterial hypertension [6, 7], the mechanisms involved in this morbidity still deserve thorough investigation. The inflammation associated with increased blood pressure manifests, systemically, increasing concentrations of circulating inflammatory markers,

such as C-reactive protein, cytokines, chemokines, and soluble adhesion molecules [8, 9]. Therefore, arterial hypertension has been considered nowadays as an inflammatory disease [10].

Epidemiological studies have revealed that periodontitis affects over 50% of the worldwide population [11], and it is likewise a major public health problem [12]. Periodontitis is a chronic inflammatory disease caused by dysbiosis of the oral microbiome and exacerbation of pro-inflammatory cytokines, including interleukin (IL)- 1-beta (IL-1β) and IL-6, and nitric oxide (NO) production, which might lead to tissue destruction [13–15]. Also, nod-like receptor family pyrin domain-containing protein-3 (NLRP3) complex inflammasome, also is increased in plasma and saliva in patients, which is associated of the progression of inflammatory and adaptative immune responses [16]. Recent studies have shown that the harmful effects of periodontitis are not restricted to the oral cavity, but can also affect the whole organism [17].

It is known that changes in arterial pressure variability (APV), as well as in heart rate variability (HRV), are predictors of morbimortality in cardiovascular diseases [18–20]. Moreover, a growing body of evidence has associated, epidemiologically, periodontal inflammation with cardiovascular diseases, such as arterial hypertension [21]. Our group recently demonstrated that periodontitis impacts the overall arterial pressure variability (APV) and cardiac performance, increases sympathetic activity and myocardial inflammation in Balb/c mice [22]. However, the effects of periodontitis in APV and HRV in hypertensive condition are not yet well established.

The inflammatory response that accompanies periodontitis has been proposed as an essential factor that may affect coronary heart disease has been associated with galectin-3 levels, involving inflammatory signaling responses [23] and blood pressure regulation [24]. It has been postulated that this relationship could be either indirect, i.e., via similar risk factors [25], or direct, through the translocation of oral bacteria into the bloodstream, causing a generalized inflammatory response [26]. Moreover, studies have indicated that hypertension accelerates the process of bone loss, which is intrinsically related to bone quality and density [27–30]. Periodontitis acting on the development and maintenance of arterial hypertension has been less consistently studied, especially regarding the hemodynamics, plasma NO levels, and inflammatory aspects.

Thus, the aim of this study was to evaluate the effect of periodontitis on HRV and APV. Moreover, to investigate, likewise, the mechanisms relating arterial hypertension and periodontitis, by evaluating the plasma levels of NO and IL-1β concentration in the myocardial tissue.

## Methods

### Animals

All experiments were performed in twelve-weeks-old male Wistar Kyoto rats (WKY) and spontaneously hypertensive rats (SHR). The rats were purchased from the Institute of Biomedical Sciences (University of São Paulo) to ensure that the experimental animals used came from the desired strain. The animals were kept on a 12-h light–dark cycle with free access to food and water. All procedures adhered to the The Animal Research: Reporting in Vivo Experiments guidelines (ARRIVE) [31]**,** and were approved by the Ethics Committee of Ribeirão Preto Medical School, University of São Paulo, São Paulo, Brazil (protocol number 218/2019).

### Experimental design

Under ketamine (50 mg/kg) and xylazine (10 mg/kg) anaesthesia, periodontitis was induced by ligation of the bilateral mandibular first molar with 4-0 sterile silk suture (Bioline, Anápolis, GO, Brazil). Control animals underwent to sham operation. After the surgery, animals received analgesic (Tramadol Hydrochloride, 12.5 mg/kg, subcutaneous). The rats were divided randomly into four groups: SHR with periodontal disease (n = 5–7, SHR + PD); SHR without periodontal disease (n = 5–7; SHR + Sham); WKY with periodontal disease (n = 6–10, WKY + PD); and WKY rats without periodontal disease (n = 6–7, WKY + Sham). Fourteen days after the initiation of periodontitis, a polyethylene catheter (Intramedic, Clay Adams, Parsippany, NJ) was implanted into the femoral artery for direct measurement of arterial pressure (AP) under ketamine (50 mg/kg) and xylazine (10 mg/kg) anaesthesia. At the following day, the arterial catheter was connected to a pressure transducer (MLT844; ADInstruments, Bella Vista, Australia) and the AP signal was amplified (ML224; ADInstruments, Bella Vista, Australia) and sampled by an IBM/PC attached to an analogue-to-digital interface (PowerLab, ADInstruments, Bella Vista, Australia). The experiments were conducted in conscious freely moving animals and AP was recorded continuously during 1 h. Following, rats were euthanised with ketamine (150 mg/kg) and xylazine (30 mg/kg) anaesthesia, and blood, jaws, and heart were collected. The jaws were then fixed with 10% formalin, and the heart was immediately frozen in liquid nitrogen. Blood samples were centrifugation at 3500 rpm for 15 min at 4 °C. The plasma was then collected, and the heart was frozen at -80 °C and stored until analysis.

### Evaluation of alveolar bone

The jaws were stained with 1% methylene blue (1 g/100 mL, diluted water) for 5 min after the remove soft tissues in 9% hypochlorite for 5 h. Following, the steps and procedures were performed as previously described [22]. Briefly, a digital camera coupled into a D.F. Vasconcellos microscope (Brazil) was used to the obtained images captured by StCamSWare 1.1. The measurement process was performed at three points on the buccal and lingual surfaces of first molar, calculating the distance (mm) between the cementoenamel junction and alveolar bone crest. The mean values in pixels were converted into millimetres using the markings of the ruler to which the jaw was attached as a reference using Image J (National Institutes of Health, USA) computer software.

### Direct pressure measurement data analysis

Pulsatile AP recordings were processed by a computer software (Blood Pressure Module for LabChart Pro, ADInstruments, Castle Hill, NSW, Australia), which employs algorithms to detect cycle-to-cycle inflection points in the pulsatile AP signal, to extract beat-by-beat time series of systolic AP and pulse interval (PI). Artefacts and ectopic beats were removed from the series if the values that exceeded 0.1- to 0.2-times the moving average of the signal. The excluded values did not exceed 1% of the overall series length [32, 33].

### Heart rate and systolic AP variability

Overall, time domain, systolic AP variability was determined by the standard deviation of successive AP values (SD) while PI variability was quantified by the standard deviation of normal-to-normal intervals (SDNN), and also by the root mean square of successive PI differences (RMSSD). These parameters were calculated in a sliding window (no overlap) of 1500 beats.

The AP and PI variability were also studied in the frequency domain by spectral analysis. PI and systolic AP beat-by-beat time series were converted to evenly spaced series at 10 Hz using cubic spline interpolation and divided in 50% overlapped segments of 512 points. The spectrum of each segment was calculated using fast Fourier transform and integrated in low- (LF, 0.2–0.8 Hz) and high-frequency bands (HF, 0.8–3.0 Hz). The median powers at LF and HF bands in absolute (abs) and normalized units (un), as well as the LF/HF ratio were estimated. Normalized values were obtained only for the PI series by calculating the proportion of LF and HF power relative to the total power of the spectrum.

Dynamic symbolic analysis, as proposed by Porta [34], was also evaluated for PI and systolic AP. The series of were converted in a sequence of symbols and evaluated in sets of three. The PI and systolic AP were uniform quantization, to the full range of values is divided into six equal levels. Each level is represented by a symbol (0 to 5), and all points within the same level were assigned the same symbol. After, sequences of three consecutive symbols were classified according to their variation pattern as zero (0 V), one (1 V), or two-unlike (2UV) variations. The data is presented in the percentage of patterns classified in each family.

### Plasma NO levels measurement

NO measurement was performed indirectly by sodium nitrate quantification chemiluminescence according to a previous study [35]. Briefly, plasma (50 μL) was deproteinized using 100 μL of absolute ethanol at 4° C for 30 min. Then, the samples were centrifuged (10.000 rpm, 10 min, 25° C) and the supernatant was used to measure nitrate nitrite using a Sievers NO Analyzer (Sievers 280 NOA; Sievers, Boulder, CO). The sodium nitrate (Sigma-Aldrich Brazil, São Paulo, Brazil) was used as a standard reference.

### IL-1β myocardial concentration measurement

Myocardial samples were homogenized in 0.5 mL of PBS and then centrifuged at 3500 rpm for 15 min at 4 °C. In the supernatants of samples were measured the IL-1β using appropriate ELISA kits (R&D Systems, Minneapolis, Minn., USA) according to the manufacturer’s instructions. The total protein was measured by the Bradford method (Bio-Rad Laboratories).

### Statistical analysis

Statistical analyses were performed using two-way analyses of variance (ANOVA), and significant differences were obtained using the Bonferroni’s multiple comparisons post-hoc test, or Kruskal–Wallis—a nonparametric statistical test—followed by Dunn’s post hoc test when the data did not pass the Shapiro–Wilk test of normality. Correlations were evaluated using Pearson correlation. Data are expressed as the mean ± standard error of the mean. For all analysis, statistical significance was considered when *P* < 0.05.

## Results

### Alveolar bone loss measurement

**Fig. 1:**
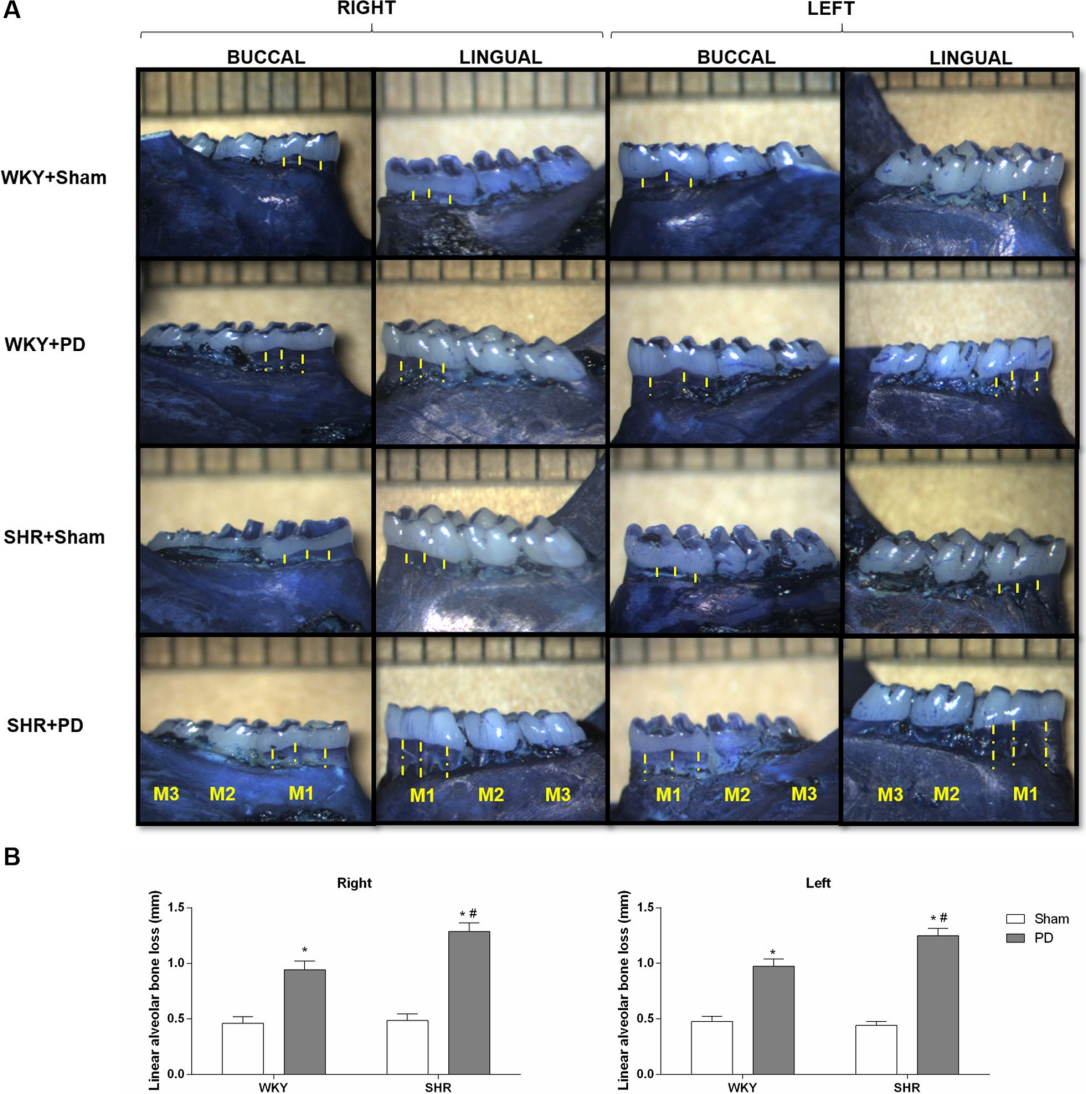
Effect of periodontitis in alveolar bone loss. (**A**) Representative images of right and left jaws of buccal and lingual surfaces of WKY rats and SHR, with and without PD. (**B**) Linear and area of the alveolar bone loss was measured macroscopically by mean of the lingual and buccal surfaces of the right and left jaws. The yellow dashed lines indicate the three distances measured in the teeth and yellow solid lines indicate alveolar bone loss area. Quantifications were performed using ImageJ 1.50i software, a version of Wayne Rasband, National Institutes of Health, USA (https://imagej.nih.gov/ij/). Data are mean ± SEM. * P < 0.05 compared to sham operated WKY. ^#^P < 0.05 compared to sham operated SHR. Wistar Kyoto rats without periodontal disease (WKY + Sham, n = 7); Wistar Kyoto rats with periodontal disease (WKY + PD, n = 10); Spontaneously hypertensive rats without periodontitis (SHR + Sham, n = 7); Spontaneously hypertensive rats with periodontitis (SHR + PD, n = 6). M1: lower first molar; M2: second lower molar; and M3: third molar

Figure 1 shows the impact of hypertension on bone alterations after PD, evaluated by three measurements of the distance between the alveolar bone crest and cementoenamel junction in the mean between buccal and lingual surfaces and right and left sides in the first molar.

In the right molar, WKY rats with PD and SHR rats with PD showed approximately 104% and 179%, respectively, higher linear bone alveolar loss than WKY without PD (*P* < 0.05). Moreover, SHR rats with PD showed 36% higher linear alveolar bone loss than WKY rats with PD (*P* < 0.05). On the other hand, no change in linear alveolar bone loss between SHR rats without PD and WKY rats without PD in both first molars.

As expected, SHR rats with PD showed approximately 163% and 28% higher distances from the cementoenamel junction to the alveolar bone crest compared to either sham-operated counterparts and WKY rats with PD, respectively, in the left first molar (*P* < 0.05).

### Hemodynamics and its variability

As expected, SHR presented higher values of mean arterial pressure (MAP) and HR (171 ± 3 mmHg and 308 ± 10 bpm) as compared to WKY rats (116 ± 5 mmHg and 317 ± 7 bpm). In WKY rats, the presence of PD did not elicit any change in either MAP (120 ± 5 mmHg) or HR (317 ± 7 bpm). On the other hand, MAP of SHR with PD was found lower (152 ± 2 mmHg) than its control counterparts. HR was similar between SHR with or without PD.

Table 1 shows variability indices of systolic AP and HR in the 4 groups of rats evaluated. Overall systolic AP variability (SD) and also the power of the LF oscillations of AP were higher in SHR as compared to WKY. However, PD did not elicit any change in systolic AP variability in either, SHR or WKY rats.

**Table 1:** Systolic arterial pressure and pulse interval indices of variability in time and frequency domains

	WKY Sham	WKY PD	SHR Sham	SHR PD
Systolic arterial pressure				
SD (mmHg)	4.8 ± 0.3	5.2 ± 0.2	8.8 ± 0.3*	6.6 ± 0.7*
LF (mmHg^2^)	2.7 ± 0.8	2.7 ± 0.4	15.8 ± 0.7*	12.3 ± 1.5*
Pulse interval				
SDNN	7.9 ± 0.9	8.5 ± 0.6	7.9 ± 0.7	7.0 ± 1.0
RMSSD	5.8 ± 0.6	6.6 ± 0.4	4.9 ± 0.2*	4.9 ± 0.8*
LF (nu)	14.3 ± 1.4	16.5 ± 2.3	22.4 ± 2.0*	20.8 ± 1.7*
HF (ms^2^)	7.3 ± 1.5	8.8 ± 0.9	6.3 ± 0.6	7.3 ± 2.3

Values (means ± SEM) of variability indices of systolic arterial pressure and pulse interval (PI) variability in time and frequency domain. SD: standard deviation of AP values; SDNN: SD of normal-to-normal values of PI; RMSSD: square root of the mean of the sum of the square of differences between successive values of pulse interval; LF and HF: power of AP or PI spectra at low- (0.2 to 0.8 Hz) and high-frequency (0.8 to 3 Hz), respectively; nu: normalized units. *P < 0.05 compared to sham operated WKY. ^#^P < 0.05 compared to sham operated SHR. Wistar Kyoto rats without periodontal disease (WKY + Sham, n = 7); Wistar Kyoto rats with periodontal disease (WKY + PD, n = 10); Spontaneously hypertensive rats without periodontitis (SHR + Sham, n = 7); Spontaneously hypertensive rats with periodontitis (SHR + PD, n = 6)

SDNN was similar among the groups; however, RMSSD was lower and LF power of PI spectra was markedly higher in SHR as compared to normotensive WKY rats. Similarly to the findings with AP variability, PD also did not cause any change in HRV in both strains of rats evaluated in this study.

### Symbolic analysis

**Fig. 2:**
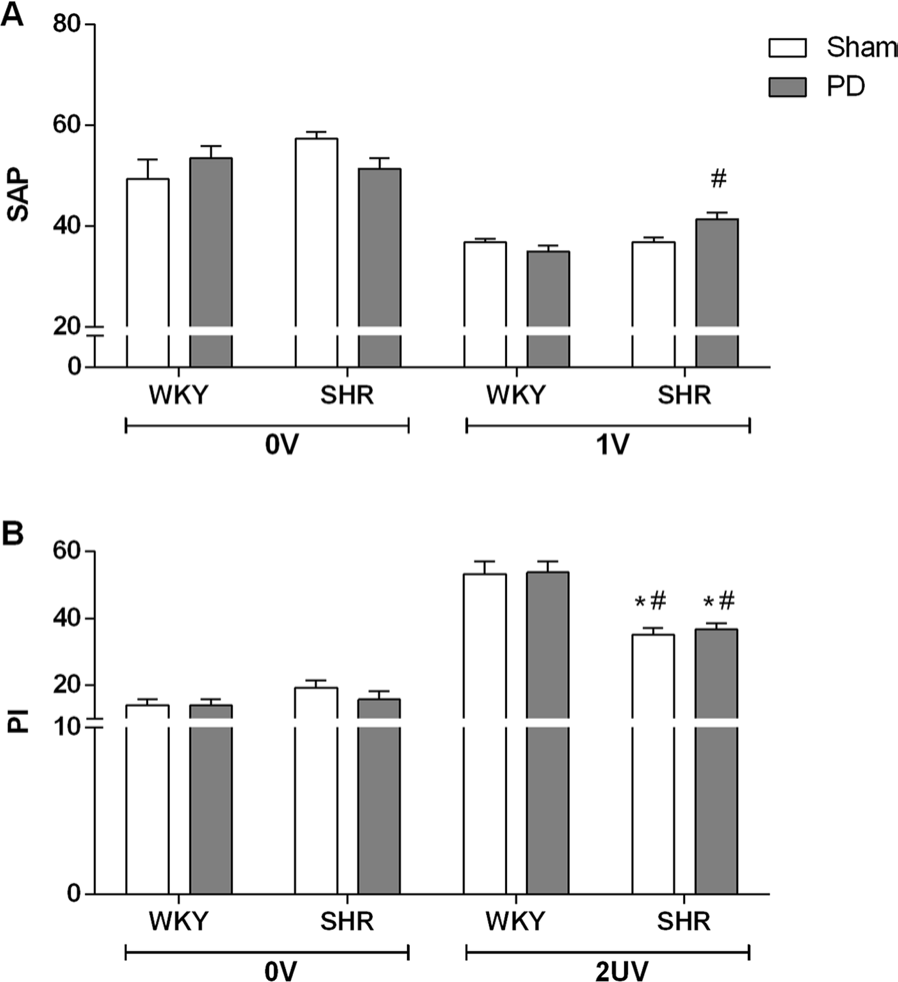
Symbolic dynamics analysis. Percentage of occurrence of families from symbolic dynamics analysis of systolic arterial pressure (**A**; 0 V and 1 V) and pulse interval (**B**; 0 V and 2UV). * P < 0.05 compared to WKY + Sham and ^#^P < 0.05 compared to WKY + PD. WKY rats without periodontal disease (WKY + Sham, n = 7); Wistar Kyoto rats with periodontal disease (WKY + PD, n = 10); Spontaneously hypertensive rats without periodontitis (SHR + Sham, n = 7); Spontaneously hypertensive rats with periodontitis (SHR + PD, n = 6)

The results of the symbolic analysis are shown in Fig. 2. 0 V pattern of systolic AP was similar among the four groups, but the percentage 1 V pattern, an index linked to sympathetic vascular modulation, was significantly higher in SHR than WKY with PD (41 ± 1% and 35 ± 1%). Nevertheless, the incidence of 0 V pattern of the IP, also an index of sympathetic cardiac modulation, was similar among all groups. The percentage of 2UV pattern of PI, a solid index of cardiac vagal modulation was found markedly lower in SHR as compared to WKY rats (42 ± 2% and 59 ± 3%). PD did not affect the incidence of 2UV pattern of HRV.

### Plasma NO and IL-1β myocardial measurements

**Fig. 3:**
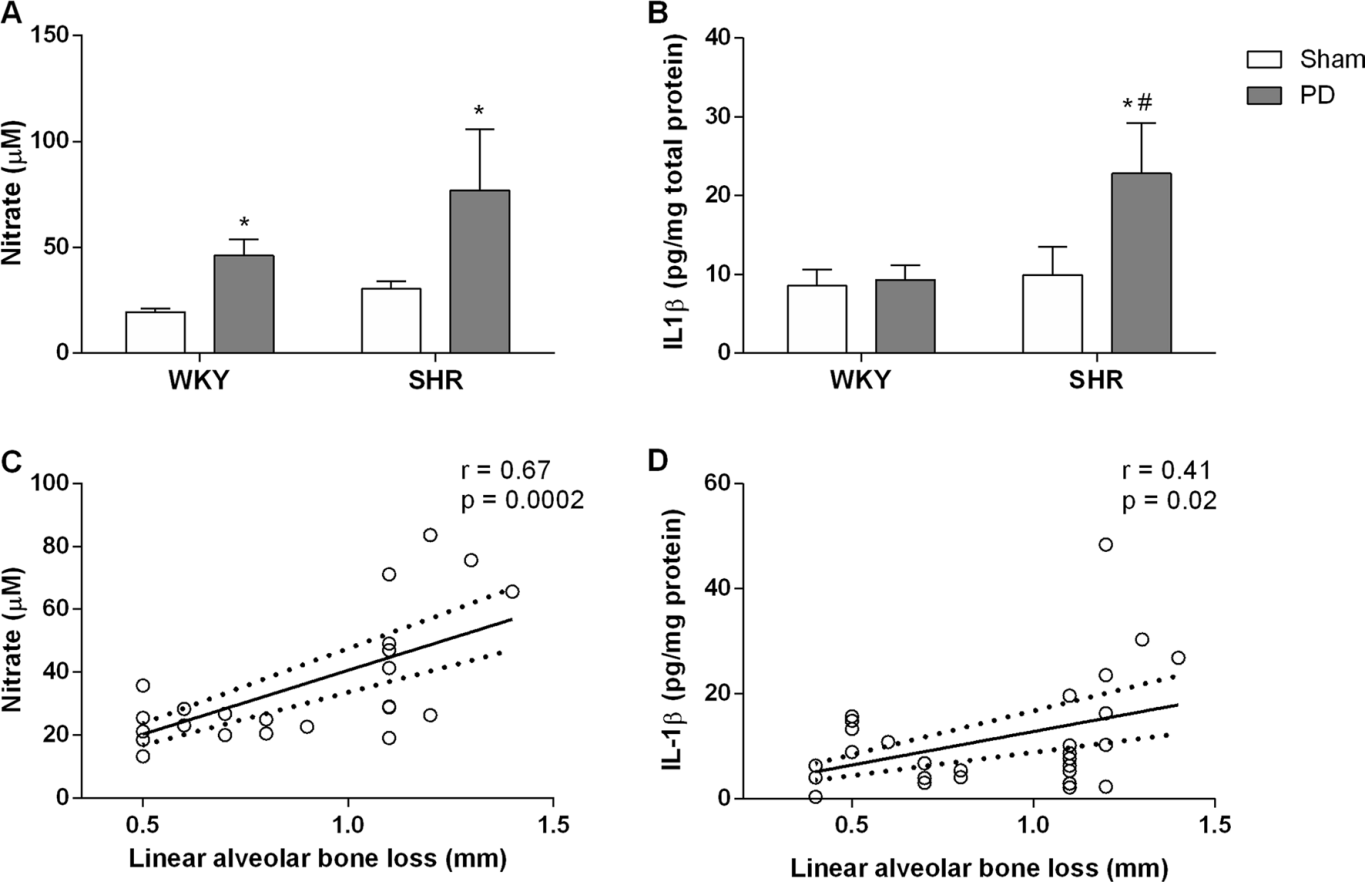
Plasma nitrate and inflammatory cytokine in the myocardial tissue. Bar Graphs show: Panel **A**, plasma nitrate level (indirect measurement of nitric oxide); Panel **B**, interleukin-1 beta (IL-1β) myocardial tissue concentration; Panel **C**, correlation between linear alveolar bone boss and plasma nitrate concentration; and Panel **D**, correlation between linear alveolar bone boss and IL-1β myocardial tissue concentration. *P < 0.05 compared to WKY + Sham and ^#^*P* < 0.05 compared to WKY + PD. Wistar Kyoto rats without periodontal disease (WKY + Sham, n = 6); Wistar Kyoto rats with periodontal disease (WKY + PD, n = 6–8); Spontaneously hypertensive rats without periodontitis (SHR + Sham, n = 5–6); Spontaneously hypertensive rats with periodontitis (SHR + PD, n = 5–7)

The plasma nitrate levels and the concentrations of IL-1β in the heart of the animals studied are presented in Fig. 3. As one can see, PD elicited a marked increase in plasma nitrate levels in both strains of rats evaluated. However, PD increased cardiac IL-1β concentrations in SHR only. This figure also shows the positive and significant relationship between mandibular bone loss and plasma nitrate or cardiac of IL-1β.

## Discussion

In the present study, the linear alveolar bone loss in jaws of SHR with PD was higher than in all other groups. AP and heart rate were higher in SHR than in their WKY counterparts. However, SHR with PD showed lower AP than SHR without PD. HRV and APV were different between SHR and WKY rats; however, no significant differences in these parameters were found between the animals with PD and their control counterparts. Also, a significant correlation was found between linear alveolar bone loss and plasma NO and myocardial IL-1β concentrations, which were higher in SHR with PD than WKY without PD. Then, short-term (15 days) experimental PD presented a decrease in AP levels in SHR, which might be due to the higher levels of plasma NO. In addition, PD induced myocardial inflammation, which can determine cardiovascular dysfunction in the future.

Ligature-induced PD is one of the most widely used experimental model in different animal species, including rats, mice, dogs and primates [36]. This model displays a body of outcomes similar to those exhibited by humans [37], i.e., local exacerbated pro-inflammatory response, junctional epithelium apical migration, and bone loss [38, 39]. Previous studies have proven that the harmful effects of ligature-induced PD are aggravated by arterial hypertension [27, 40]. The arterial hypertension enhances bone loss and impairs bone repair [27, 40]. In line with these studies, in the current study, PD increased alveolar bone loss in SHR. Nevertheless, the impact of PD in the hemodynamics (APV and HRV) of a hypertensive condition is poorly understood.

Previous studies showed that the impact of PD is not restricted to the oral cavity, but can be associated with systemic diseases, such as arterial hypertension, myocardial infarction, stroke, and atherosclerotic vascular disease [21, 25, 41, 42]. Moreover, our laboratory showed that 30 days of ligature-induced PD in mice decreased the cardiac function (reduction of ejection fraction and cardiac output) and induced autonomic imbalance characterized by sympathetic overactivity [22]. Patterns of variability of cardiovascular parameters (HRV and APV) are well documented, and being widely used as predictors of morbidity and mortality in different cardiovascular and systemic diseases, at clinical and experimental conditions [23, 24]. However, to our knowledge, this is the first experimental study that evaluated the impact of PD on cardiovascular variability in hypertensive rats, particularly in the SHR.

In order to examine the effects of PD on a hypertensive condition, the SHR was chosen as the experimental model. SHR is one of the most widely-used genetic models of arterial hypertension, because it is considered the experimental model of human essential hypertension [43]. Apropos, the SHR show systemic manifestations similar to the human disorder, such as hemodynamic and endocrine outcomes [43]. Moreover, the normotensive control counterparts used in the current study were the WKY rats, because these animals have the same genetic background of the SHR, without exhibiting a spontaneous increase in arterial pressure [43]. In fact, in this study, the SHR showed, as expected, higher AP and HR levels when compared to their WKY counterparts. Moreover, the SHR also showed higher values of the overall APV, AP spectra at the low-frequency (LF) band, and incidence of 1 V sequences from the symbolic analysis. Also, the SHR showed low overall HRV, low indices of HRV linked to parasympathetic cardiac modulation (HF power of HR spectra and RMSSD), and high indices of HRV related to sympathetic activity (LF power of HR spectra). These results are in line with previous studies, which demonstrated sustained sympathetic overactivity, and decreased parasympathetic modulation in SHR, compared to WKY [44–48].

Nevertheless, the presence of PD did not affect the variability indices of HR and AP in neither WKY nor SHR. These findings may be related to the short-term development (15 days) of PD since our laboratory showed an impact of PD in APV indices only after 30 days of dental ligature in mice [22]. However, surprisingly, the SHR with PD showed a lower AP than their counterparts submitted to Sham surgery. Given that ligature-induced PD and SHR rats do not reproduce all aspects of human disease and the current study used a short time frame of investigation, the results must be cautiously interpreted and carefully extrapolated to a clinical context. Nevertheless, a possible mechanism for this finding might be associated with the higher plasma levels of NO-induced PD. NO is a short living product synthesized from L-arginine by a family of enzymes: Type 1-NO synthase-neuronal enzyme; Type 2-NO synthase-inducible enzymes (iNOS) found in macrophages; and Type 3-NO synthase—endothelial cell enzymes (eNOS) [49]. NO is an endothelial-derived relaxant of the vascular smooth muscle and an inflammatory mediator, regulating vascular homeostasis [50]. The overproduction of NO by active macrophages induces nonspecific immunity and cytotoxic effect against invading microbial pathogens triggered by PD [49]. Moreover, the high levels of NO increase the damage of cellular proteins and lipids, leading to PD progression [49]. Previous studies also demonstrated that animals chronically treated with NO synthase inhibitors develop arterial hypertension, characterized by endothelial dysfunction [51, 52].

Recently, PD has also been associated with transient endothelial dysfunction [53–55] and increased levels of plasma NO [56]. Clinical and experimental studies have discussed the role of NO in the progression of PD. The increase of NO is not restricted to the oral cavity, but some studies showed that patients with periodontitis had increased plasma concentration of NO (nitrate and nitrite) [56, 57]. In fact, the results of the current study showed a significant increase of plasma NO levels, which were correlated with the development of local alveolar bone loss in PD and the short-term hypotensive response. This result is in line with the previous study that showed a role for NO associated with the severity of human periodontal disease [58]. Nevertheless, Menaka and co-workers revealed that individuals with PD had significantly higher NO levels in the serum than healthy subjects [56]. Moreover, Lohianai and co-workers showed that the augmented production of NO played a significant role in the pathogenesis of PD [59]. Besides, Herrera and co-workers demonstrated that NO contributes to the physiopathogenesis of PD since the concomitant bone loss is significantly reduced in L-NAME treated rats [60]. On the other hand, Higashi and co-workers showed that PD impairs endothelium-dependent vasodilation in both healthy and hypertensive young men, by decreasing NO availability [54]. Although the available information on NO production during PD is still controversial, the current study corroborates with the notion that there is an increase of NO release in response to PD. Thus, the NO overproduction observed in the present study would justify the hypotensive response in SHR.

New insights have been reported that galectin-3 and NLRP3 increase in PD patients and play a role in dysbiosis's inflammatory and immune response [16, 23]. Galectin-3 has been demonstrated to be implicated during the host defense against microbes, while NLRP3 induces up-regulation of IL-1β, a proinflammatory cytokine-mediated by inflammasome activation [16, 23]. Moreover, IL-1β increases tissue destruction, osteoclast formation, and bone resorption. These functions affect neutrophil chemotaxis and activation, and endothelial cell function. In fact, chronic inflammation caused by PD is involved in systemic effects in distant organs, such as the heart [61–65]. In the current study the hearts from SHR with PD showed increased pro-inflammatory cytokine IL-1β concentrations. Herrera and co-workers suggested that NO mediates the systemic effects of PD on myocardial tissue, which may be due to the transient bacteremia or the immunological activation caused by the dysbiosis of oral microorganisms in the ligature-induced PD rats [60]. The presence of IL-1β is strongly associated with cardiovascular diseases, which could cause a long-term derangement of the heart and autonomic function; while its inhibition decreases AP, arterial hypertension and residual inflammatory risk [9, 66]. Thus, further studies are needed to elucidate the role of IL-1β induced by long-term PD in the modulation of the autonomic nervous system and the AP response.

## Conclusion

In conclusion, the current study's data support the notion that PD increases the NO levels in the blood, which could affect the AP in SHR. However, PD did not affect either HRV or APV. In addition, the observations of the present study strongly suggest that the inflammation induced by PD in first molars can lead to an increase in IL-1β in myocardial tissue. Further studies should be conducted to evaluate the cardiovascular function in long-term experimental PD in SHR.

## Data Availability

The datasets analysed during the current study are available from the corresponding author on reasonable request.

## Abbreviations

PD: Periodontal disease
NO: Nitric oxide
AP: Arterial pressure
APV: Arterial pressure variability
NLRP3: Nod-like receptor family pyrin domain-containing protein-3
HRV: Heart rate variability
SHR: Spontaneously hypertensive rats
WKY: Wistar-Kyoto rats
